# Superior removal of arsenic from water with zirconium metal-organic framework UiO-66

**DOI:** 10.1038/srep16613

**Published:** 2015-11-12

**Authors:** Chenghong Wang, Xinlei Liu, J. Paul Chen, Kang Li

**Affiliations:** 1 NUS Graduate School for Integrative Sciences & Engineering (NGS), National University of Singapore, Singapore, 117456, Singapore; 2 Department of Civil and Environmental Engineering, National University of Singapore, Singapore, 117576, Singapore; 3 Department of Chemical Engineering, Imperial College London, London, SW7 2AZ, United Kingdom

## Abstract

In this study, water stable zirconium metal-organic framework (UiO-66) has been synthesized and for the first time applied as an adsorbent to remove aquatic arsenic contamination. The as-synthesized UiO-66 adsorbent functions excellently across a broad pH range of 1 to 10, and achieves a remarkable arsenate uptake capacity of 303 mg/g at the optimal pH, i.e., pH = 2. To the best of our knowledge, this is the highest arsenate As(V) adsorption capacity ever reported, much higher than that of currently available adsorbents (5–280 mg/g, generally less than 100 mg/g). The superior arsenic uptake performance of UiO-66 adsorbent could be attributed to the highly porous crystalline structure containing zirconium oxide clusters, which provides a large contact area and plenty of active sites in unit space. Two binding sites within the adsorbent framework are proposed for arsenic species, i.e., hydroxyl group and benzenedicarboxylate ligand. At equilibrium, seven equivalent arsenic species can be captured by one Zr_6_ cluster through the formation of Zr-O-As coordination bonds.

Arsenic contamination is a global threat due to its toxicity and carcinogenicity^1^. Typical arsenic concentration in contaminated groundwater ranges from 0.5 to 2.5 ppm, and much higher (usually >100 ppm) in industrial waste water^2^. Exposure to arsenic-polluted water would result in such severe health problems as liver, lung, kidney, and skin cancers^1^,^2^,^3^. Hence, arsenic has been categorised by World Health Organization (WHO) as the first priority issue among the toxic substances^4^. Although aquatic arsenic possesses different oxidation states, the inorganic arsenic is usually oxidised to arsenate As(V) in various water bodies. Due to the high mobility of arsenate species in water streams as well as its ease in accumulation in human body and food chain^5^, effective removal of aquatic arsenate has been an important topic in water treatment.

Adsorption is considered as one of the most promising techniques for wastewater decontamination owing to the high efficiency, low cost and ease in operation^6^. Intensive studies have been carried out to develop various adsorbents for arsenic removal and some commercial adsorbents are as well available^6^,^7^,^8^. Despite that, the arsenic adsorption capacity of conventional adsorbents like activated carbons^6^, activated alumina^9^ and powdered zeolite^10^ is unsatisfactory. In order to further improve the adsorption capacity, strategic methods including reducing the particle size of adsorbents or preparing materials with hierarchically ordered structures were employed^11^,^12^,^13^,^14^. These approaches may increase the surface area of adsorbent for efficient contact, however they could complicate the synthesis process and consequently raise the production cost. Moreover, although a few recently reported adsorbents exhibited enhanced adsorption capacity, such as γ-Fe_2_O_3_ nanoparticles embedded silica and yttrium–manganese binary composite^15^,^16^, their applicable pH ranges are quite limited. Hence, adsorbents with better performance are on demand for arsenic decontamination from water.

Metal-organic frameworks (MOFs), a new class of hybrid porous materials built from organic linkers and inorganic metal (or metal-containing cluster) nodes through coordination bonds^17^, have attracted tremendous attention in recent years^18^. Benefitting from their versatile architectures and customizable chemical functionalities, MOFs have been widely applied in gas storage, sensing, catalysis, separation, etc^19^,^20^,^21^,^22^,^23^. However, the hydrothermal stability of MOFs remains a challenge as most MOFs are sensitive to water^24^; very few of them stay chemically stable in an acidic or basic aqueous solution^25^. This restricts the practical applications of MOFs in water treatment. Recently, some water stable MOFs have been developed and applied for heavy metal ions decontamination^26^,^27^,^28^,^29^,^30^,^31^,^32^. In particular, ZIF-8, MIL-53 and Fe-BTC MOF materials were put into aquatic arsenic removal tests^30^,^31^,^32^, but no outstanding performance was observed in comparison with other commercial and synthetic adsorbents.

Since zirconium based adsorbents such as amorphous zirconium oxide nanoparticles^33^ and zirconium immobilized nano-scale carbon^34^ demonstrated strong affinity towards arsenic species, a porous crystalline material containing zirconium, which provides a larger contact area and more active adsorption site, may deliver a better arsenic uptake performance. Recently, a series of zirconium MOFs (Zr-MOFs) with exceptional chemical and thermal stability has emerged^35^,^36^,^37^,^38^,^39^,^40^. UiO-66 framework (UiO stands for University of Oslo) is one prototypical Zr-MOF^35^, constructed with Zr_6_O_4_(OH)_4_ clusters and terephthalate (1,4-benzenedicarboxylate, BDC) linkers. As shown in Fig. 1(a), the octahedral cluster of UiO-66 contains six-centred Zr cations, as well as eight μ3-O bridges, four of which are protonated. Moreover, each cluster unit is connected to 12 neighbouring clusters by BDC linkers to establish an expanded face-centred-cubic (**fcu**) arrangement, as shown in Fig. 1(b). The high degree of topological connectivity together with the strong coordination bonds between zirconium and oxygen renders UiO-66 to be greatly hydro-stable, even under acidic or some alkaline conditions^35^. This provides a theoretical basis of applying UiO-66 in water treatment. Thus far, a few researchers have employed UiO-66 framework to capture contaminants in water solution^27^,^41^,^42^,^43^,^44^, but no reports appeared in any journals on arsenic removal.

In this study, water stable Zr-MOF (UiO-66) with micron particle size was synthesized and applied as an adsorbent to uptake arsenic species, specifically aquatic arsenate As(V). To the best of our knowledge, this is the first work of applying Zr-MOF in arsenic pollutant removal from water. Proper characterizations, adsorption studies and mechanism analyses were carried out to examine the arsenic adsorption performance of UiO-66 adsorbents. pH applicable range and adsorption capacity as ones of the key operational parameters were assessed in detail. The adsorbent structures as well as adsorption mechanisms were studied by analysing the scanning electron microscopy coupled with energy-dispersive X-ray spectroscopy (SEM-EDX), powder X-ray diffraction (PXRD) and Fourier transform infrared spectroscopy (FTIR). This study unveils the excellent performance of UiO-66 adsorbent in arsenic removal from water, which would provide significant new insights to the application of MOFs in water treatment and lead to an advanced adsorbent material in arsenic decontamination industry.

## Results and Discussion

### Characterization of adsorbent

**Figure 2:**
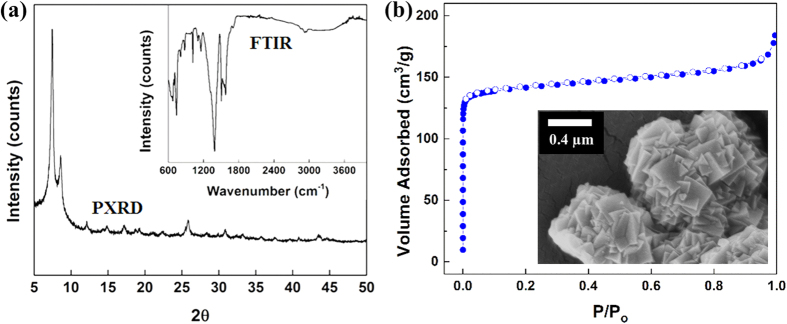
(**a**) PXRD pattern and FTIR spectrum of pristine UiO-66 adsorbent. (**b**) Nitrogen adsorption (filled circles)-desorption (open circles) isotherms and SEM image of pristine UiO-66 materials.

The PXRD pattern as well as FTIR spectrum of as-synthesize UiO-66 materials is shown in Fig. 2(a). It can be observed that the main XRD peaks and the IR bands matched well with those in literature^35^. Representative vibrations like peaks at 1590 and 1390 cm^−1^ associated to the carboxylate groups and peaks at 730 and 680 cm^−1^ corresponding to Zr-(μ3)O can all be observed in the FTIR spectrum^35^. The characterization data indicate that the UiO-66 framework has been successfully prepared. The surface morphology of UiO-66 adsorbents is presented in Fig. 2(b). The UiO-66 materials were in micron particle size, and the crystals were well intergrown with sharp edges. Besides, the Brunauer–Emmett–Teller (BET) surface area of UiO-66 was calculated to be 569.6 m^2^/g, based on the N_2_ adsorption–desorption isotherms at 77 K, as shown in Fig. 2(b).

### Arsenate adsorption

#### pH effect

**Figure 3:**
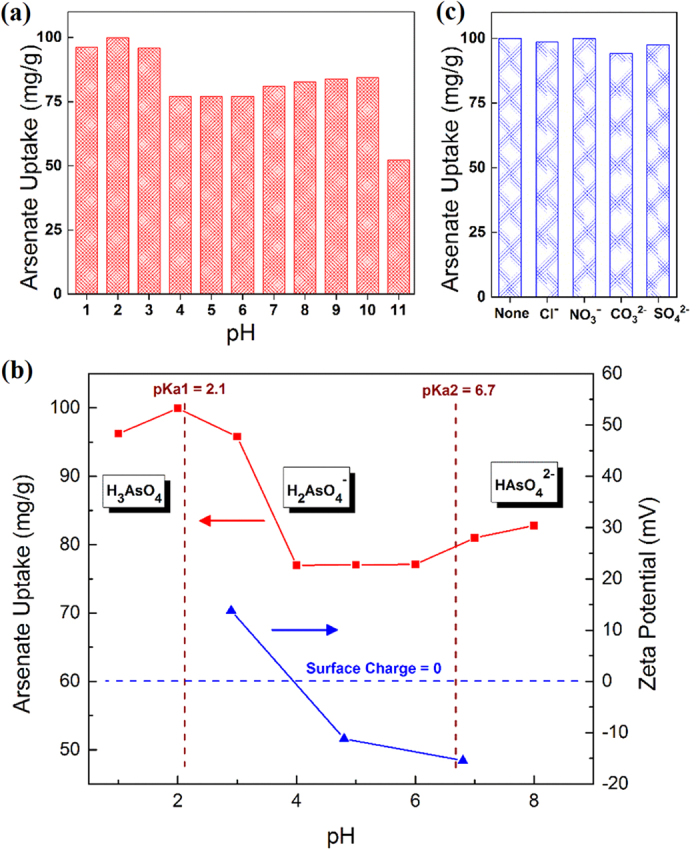
(**a**) pH effect on arsenate adsorption. (**b**) pH effect on As(V) speciation, adsorbent surface charge and adsorption performance. (**c**) Coexisting anion effects on arsenate adsorption at pH 2. [UiO-66] = 0.5 g/L, [As(V)]_0_ = 50 mg/L, [coexisting anions] = 1 g/L, T = 25 ± 1 °C.

pH value is one of the key operational parameters in practical water treatment, as it may influence both the adsorbent structure and the distribution of pollutant species. The pH effect on the arsenate removal process using UiO-66 adsorbents was investigated and shown in Fig. 3(a). The UiO-66 adsorbent demonstrated an outstanding arsenate uptake efficiency across a very broad pH range of 1 to 10. With the initial arsenate concentration of 50 ppm, the adsorbents can accomplish generally more than 75 mg/g decontamination performance in this pH range. Moreover, at very acidic conditions of pH 1 to 3, more than 95 mg arsenate can be removed by one gram of UiO-66 adsorbents; especially at pH 2, the best adsorption performance of nearly 100 mg/g was achieved. Further increasing the water pH to 11, however, the adsorption performance decreased considerably to 52 mg/g. This could be due to the onset of structural decomposition of UiO-66 under too basic condition^45^.

To better understand the relationship between water pH and adsorbent performance, zeta potential as well as arsenate speciation analyses were conducted and illustrated in Fig. 3(b). The point of zero charge was identified to be pH = 3.9, which indicates a positively charged outer surface of UiO-66 adsorbent when pH is below 3.9 and a negatively charged outer surface when pH is above 3.9. In addition, the predominant species of arsenate in water bodies exist as: H_3_AsO_4_ at pH below 2.1, H_2_AsO_4_^−^ at pH from 2.1 to 6.7, and HAsO_4_^2−^ at pH from 6.7 to 13.4, respectively. It can be found that electrostatic interaction played a certain role in the adsorption process, e.g., at pH 3 anionic arsenate species could be effectively attracted to the proximity of positively charged adsorbents, which resulted in a better adsorption performance compared to those when pH is higher than 3.9. However, electrostatic interaction did not solely control the adsorption process, since the best arsenate uptake performance appeared at pH 2 where the dominant arsenate species (H_3_AsO_4_) present as zero valence and deliver no electrostatic attraction. The proposed adsorption mechanism (as discussed in *Section Adsorption mechanism*) suggests that arsenic species were bound to the UiO-66 adsorbents through two coordination processes, which are similar to an acid-base interaction. Thus, in spite of electrostatic force, the increasing abilities of arsenate species (H_3_AsO_4_) to release H ions and bind to the hydroxyl sites in UiO-66 adsorbents at very acidic conditions (pH 1–2) significantly facilitate the arsenic uptake process, which resulted in the best adsorption efficiency in this pH range.

In addition, it should be noticed that, with the initial arsenate concentration of 50 ppm, the arsenate decontamination performance at pH 7 is more than 80 mg/g. The decent arsenate uptake efficiency of UiO-66 adsorbent at neutral pH favours its application in the remediation of surface and ground contaminated water that are normally associated with a neutral pH condition (pH = 7 ± 1). Furthermore, arsenic contaminated industrial wastewater normally varies in pH and contains different coexisting ions^46^,^47^. As shown in Fig. 3(a,c), the UiO-66 adsorbent could effectively capture arsenic across a broad pH range (1–10), and its arsenic uptake capability can hardly be inhibited by some commonly coexisting anions. Less operational cost is required, as any pre-treatment or additional pH adjustment steps could be avoided. Therefore, UiO-66 is considered as a promising arsenic adsorbent for industrial wastewater treatment.

#### Adsorption isotherm

**Figure 4:**
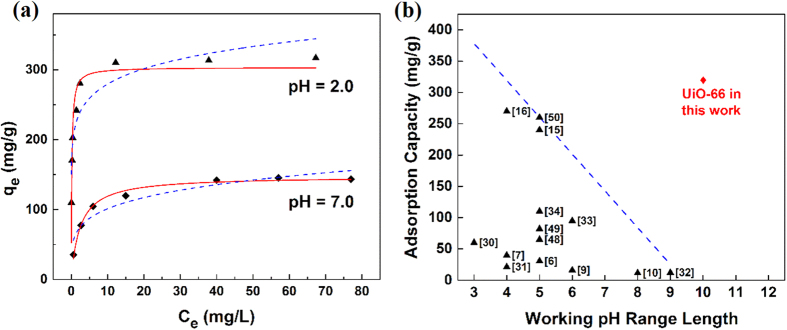
(**a**) Adsorption isotherms of arsenate onto the UiO-66 adsorbent at pH = 2 and 7; Langmuir fitting model is in red solid lines, Freundlich fitting model is in blue dash lines; [UiO-66] = 0.5 g/L, T = 25 ± 1 °C. (**b**) Comparison on arsenic adsorption performance among prevalent adsorbents. This figure was made based on Table 2; working pH range length is defined as how many integral pH values the working pH range covers.

The arsenate adsorption isotherms of UiO-66 were studied at pH 2 and 7. pH 2 was opted as it is the optimal condition at which the UiO-66 adsorbent could perform the best; neutral pH 7 was also selected to represent most natural water. The experimental results together with both Langmuir and Freundlich fitting lines are plotted in Fig. 4(a), and the best fitted parameters are summarised in Table 1. The comparatively higher correlation coefficients (*r*^*2*^) of Langmuir model indicates a monolayer adsorption process in this case. Besides, the arsenate adsorption capacity of UiO-66 adsorbent, according to the Langmuir isotherms, is as high as 303.34 mg/g and 147.71 mg/g at pH 2 and 7, respectively.

Compared to previously reported adsorbents shown in Table 2 and Fig. 4(b), the UiO-66 adsorbent delivers the best arsenic adsorption capacity, much higher than that of commercial adsorbents (approximately 50 mg/g)^7^,^8^ and synthetic adsorbents (5–280 mg/g, generally less than 100 mg/g)^6^,^9^,^10^,^15^,^16^,^30^,^31^,^32^,^33^,^34^,^48^,^49^,^50^. Most prevalent adsorbents can seldom achieve 100 mg/g even at optimal pH. A few recently developed adsorbents, e.g., γ-Fe_2_O_3_ embedded silica and yttrium-manganese binary composite, exhibited satisfactory arsenic adsorption capacity of more than 200 mg/g. However, their synthesis methods are quite complicated and costly, and their working pH ranges are rather limited. With reference to the highest adsorption capacity, the broadest pH applicable range, as well as the relatively facile method for scalable synthesis^20^,^51^,^52^, the UiO-66 adsorbent is regarded as a prospective material for arsenic removal from water.

**Figure 5:**
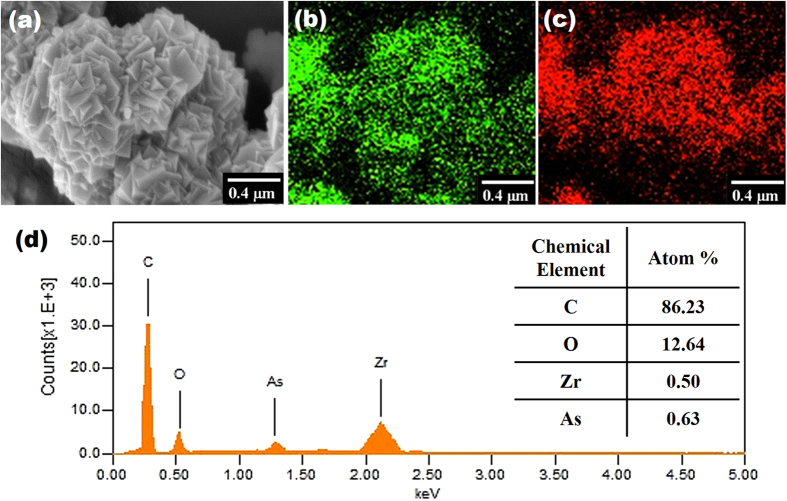
SEM image (**a**) and corresponding EDX data (b–d) of UiO-66 sample. The green and red signals in (**b**) and (**c**) represent Zr and As, respectively. The quantitative composition of C and O in (**d**) is not accurate as the carbon tape was employed as background.

Moreover, the used UiO-66 samples after adsorption tests at optimal pH were examined by SEM-EDX. It can be clearly observed in Fig. 5 that the framework morphology was reserved after the adsorption process. The elemental mapping of used adsorbents verifies the presence of arsenic species within the UiO-66 framework. Furthermore, the quantitative elemental analysis suggests that the molecular ratio between Zr and As is approximately 6 to 7.5, based on which the uptake of arsenic by UiO-66 adsorbents can be calculated. As the chemical formula of UiO-66 is Zr_6_O_4_(OH)_4_(CO_2_C_6_H_4_CO_2_)_6_, one gram of UiO-66 is equivalent to (1/1662 = 0.60) mmol. Approximately, one UiO-66 cluster containing six Zr atoms could capture seven As species. Thus, one gram of UiO-66 should be able to capture (0.60*7 = 4.20) mmol As, which is equivalent to (4.20*75 = 315) mg. This value agrees well with the isotherm analysis result that specifies an arsenic adsorption capacity of 303.34 mg/g.

### Adsorption mechanism

**Figure 6:**
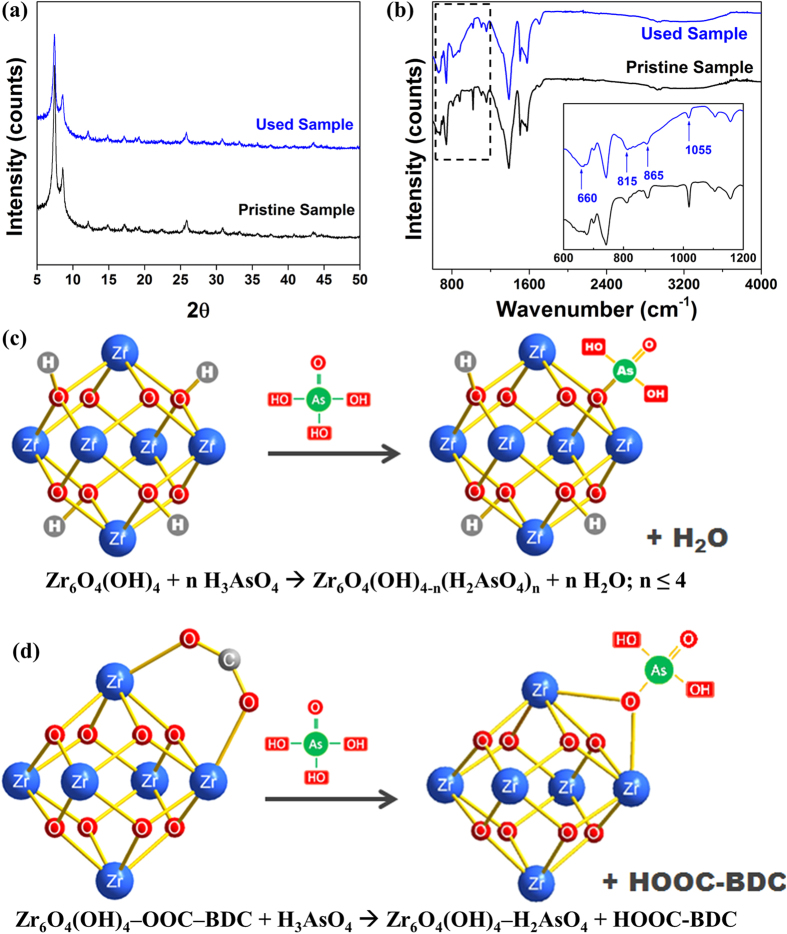
PXRD patterns (a) and FTIR spectra (**b**) of UiO-66 samples before and after use. In (**b**), the spectra from 600–1200 cm^−1^ is enlarged in the lower right corner. Proposed adsorption mechanism of arsenate onto UiO-66 through coordination at (**c**) hydroxyl group and (**d**) BDC ligand. In (**d**), H atoms in the cluster are omitted for clarity; **(OOC)** is part of the BDC linker **(-OOC-benzene-COO-)** that is linked to another Zr_6_ cluster.

To better understand the mechanism of arsenate adsorption on the UiO-66 adsorbent, PXRD and FTIR experiments were conducted to characterize the used materials, as shown in Fig. 6(a,b). No change was found in the PXRD patterns before and after adsorption, as all the characteristic peaks are present without the rise of any new peaks. This confirms the good stability of UiO-66 framework throughout the test and no damage of the crystal structure. Furthermore, compared the FTIR spectrum of used UiO-66 sample to that of the pristine material, a significant new band centred at 830 cm^−1^ appeared. The 815 cm^−1^ peak corresponding to the Zr−O−As group^53^ proves the binding of arsenic onto UiO-66 adsorbents. Moreover, the peak rising at 865 cm^−1^ is related to the combination of both symmetric and asymmetric stretching vibrations of the As–O bond^34^. In addition, a small peak at 660 cm^−1^ is identified, which would be due to the presence of As–OH asymmetric stretching^34^. The above findings confirm the formation of arsenic complexes within UiO-66 framework via establishing Zr-O-As coordination bonds.

In a unit cell of UiO-66 framework, there are two different Zr-O linkages: one is Zr-O(μ3)-Zr bridge in between Zr centres, and the other is Zr-O-C connection between Zr and BDC linkers. As reported^50^,^54^, the hydroxyl groups on adsorbent (e.g., metal oxides) surface are primarily responsible for the adsorption of arsenic. Moreover, it can be found that the peak at 1055 cm^−1^ related to the bending vibrations of hydroxyl groups on metal oxide clusters (Zr–OH)^33^ became much less obvious after adsorption, as shown in Fig. 6(b). Thus, the first likely adsorption site on UiO-66 is the μ3-O, specifically the protonated oxygen connecting to Zr, which provides four Zr-OH groups in a unit Zr_6_ cluster to attract maximum four equivalent arsenate species. As illustrated in Fig. 6(c), the arsenate species, e.g., H_3_AsO_4_, acted as acid binding to the hydroxyl groups in Zr-containing clusters, after which the releasing H ions and hydroxyl groups formed water to maintain charge balance in the solution. Furthermore, the molar ratio between Zr and As in the used UiO-66 adsorbent was found to be around 6:7 (isotherm study in *Section Adsorption isotherm*), which implies another possible adsorption site existing in the UiO-66 framework, i.e., Zr-O-C connection between Zr and BDC. The adsorption could take place by exchanging some BDC ligands with arsenate species as illustrated in Fig. 6(d). The adsorption induced hydroxyl and BDC ligand exchanges would lead to the formation of arsenic complexes in the UiO-66 framework, while the aforementioned coordination processes did not disintegrate the main crystal structure of UiO-66 adsorbent. The framework remained intact throughout the test according to the PXRD results shown in Fig. 6(a).

Furthermore, compared to nanoparticle adsorbents in Table 2, Zr-MOF (UiO-66 in this study) performs better in adsorption attributed to the specific structural features, i.e. 3D porous framework containing zirconium oxide clusters. Conventional nanoparticles are generally associated with non-accessible bulk volume, of which the active sites are only present on outer surface^32^. Amorphous nanoparticles with irregular porous structures may provide larger contact areas and more active sites, but the improvement is restricted^33^. Generally, strategic methods to enlarge the adsorbent’s surface area and consequently improve adsorption performance include reducing the particle size and preparing hierarchically ordered materials or core shell materials^11^,^12^,^13^,^14^. However, these approaches would complicate the adsorbent synthesis process and substantially increase the production cost. MOF, as a highly porous host material with regular crystallinity, renders a large contact area for the diffusion and interaction of pollutant species. Howarth and co-workers^27^ reported that Zr-based MOFs are effective for selenium remediation; NU-1000 in particular provided the highest adsorption capacity and fastest uptake rate towards aqueous selenium compounds, owing to the large apertures and substantial numbers of node-based adsorption sites. With regard to the UiO-66 adsorbent developed in this study, arsenic as pollutant species could attach to seven active sites in one unit cluster and the dimension of one unit cluster is less than unit nanometre^35^. This exposes more active sites on the UiO-66 adsorbent to coordinate with arsenic species compared to most conventional nanoparticles in unit space.

## Conclusions

In this study, water stable Zr-MOF (UiO-66) with particle size in micrometre order was synthesized and applied as an adsorbent to uptake arsenate species. To the best of our knowledge, this is the first work of applying Zr-MOF in arsenic pollutant removal from water. The UiO-66 adsorbent functioned excellently across a broad pH range, from very acidic 1 to basic 10, with the best adsorption performance at pH 2. The presence of some common anions had little influence on the arsenic adsorption process. Furthermore, the UiO-66 adsorbent achieved a remarkable arsenate uptake capacity of 303.34 mg/g at the optimal pH. This is the best arsenate adsorption capacity ever reported, much higher than that of other commercial and synthetic adsorbents (5–280 mg/g, generally less than 100 mg/g). The mechanism study proposed two binding sites within the adsorbent framework for arsenic species, i.e., hydroxyl group and BDC ligand. At equilibrium, seven equivalent arsenic species can be captured by one Zr_6_ cluster through the formation of Zr-O-As coordination bonds. To conclude, this study provides significant new insights to the application of MOFs in water treatment. The enhanced adsorption capacity of UiO-66 adsorbent compared to most conventional nanoparticle adsorbents was due to the highly porous structure containing zirconium oxide clusters, which provides a larger contact area and more active sites in unit space. With the superior adsorption performance towards aquatic arsenic species, UiO-66 could work as a promising advanced adsorbent in the arsenic decontamination industry.

## Methods

### Materials

Unless otherwise stated, all the chemicals were used as received without further purification. The reagents including zirconium(IV) chloride (ZrCl_4_, 99.5%), 1,4-benzenedicarboxylic acid (BDC, 98%), and sodium arsenate dibasic heptahydrate (Na_2_HAsO_4_•7H_2_O, 98%) were purchased from Sigma-Aldrich. Moreover, ethanol (99.9%), dimethylformamide (DMF, 99.9%), sodium nitrate (99%), sodium chloride (99%), sodium sulfate, anhydrous (99%), sodium carbonate anhydrous (99.8%), nitric acid (68%), and sodium hydroxide (99%) were purchased from VWR. The stock solution of 1000 mg/L arsenate was obtained by dissolving Na_2_HAsO_4_•7H_2_O in 1 L deionized (DI) water (Analytic lab, ACEX, Imperial College London). The solutions of required concentrations used in this study were prepared by diluting the arsenate stock solution with DI water. pH adjustment was conducted using nitric acid or sodium hydroxide.

### Synthesis of UiO-66

UiO-66 was prepared based on the procedure described by Cavka *et al.*^35^, with some modifications. ZrCl_4_, BDC and H_2_O were dissolved in DMF under stirring according to a specific molar composition: Zr^4+^/BDC/H_2_O/DMF = 1:1:1:500. The solution was then transferred to Teflon-lined stainless steel autoclaves and heated at 120 °C for 48 h in a convective oven (UF30, Memmert). Afterwards, the autoclaves were cooled down to room temperature. The UiO-66 powders were washed by ethanol with the assistance of centrifuge (Thermo Scientific Legend X1R) and dried at 120 °C overnight under vacuum condition (Fistreem Vacuum Oven) for further use.

### Characterizations of UiO-66

The surface morphology of the UiO-66 adsorbent was studied by using a scanning electron microscope (SEM, LEO Gemini 1525) coupled with Energy-dispersive X-ray (EDX). Moreover, the crystal structure of adsorbent was analysed by a powder X-ray diffractometer (PXRD, Panalytical Xpert). The X-Ray diffractometer is operated with Ni-filtered Cu Kα radiation at a voltage of 40 mV and a current of 40 mA. To be ready for XRD study, the samples were dried at 120 °C overnight under vacuum condition. Furthermore, the Fourier transform infrared (FTIR) spectrum was employed to study the structure characteristics of samples and determine the vibration frequency changes due to the adsorption process. The adsorbent materials before and after adsorption were analysed by a FTIR spectrometer (Spectrum 100, PerkinElmer) equipped with diamond ATR (attenuated total reflection) crystal. In addition, the surface charges of UiO-66 adsorbents at different pH were measured by a zeta potential analyser (ZetaPALS, Brookhaven Instruments), in order to identify the point of zero charge (PZC); the specific surface area of adsorbent was determined by N_2_ adsorption–desorption isotherms which was measured by gas adsorption analyser instrument (3Flex, Micrometrics) at 77 K. In particular, the used UiO-66 samples after arsenic adsorption were collected using centrifuge and then washed thoroughly with DI water before drying in the vacuum oven for proper characterization.

### Arsenate adsorption experiments

The adsorption tests were investigated at room temperature (25 ± 1 °C). In the pH effect experiment, a series of 50 mL arsenate solutions with initial concentration of 50 ppm was prepared using the stock solution. UiO-66 adsorbents with a dosage of 0.5 g/L were added into the solutions that were going to be constantly shaken with the rate of 220 rpm. The solution pH ranging from 1 to 11 was respectively controlled throughout the test. The pH of solutions was measured by an ORION 525A pH meter. According to the preliminary experiment, the adsorption reaches equilibrium within 48 hours. Hence, after 48 hours of contact time, the solutions were then filtered through a 0.45 mm filter and the residual arsenic concentration of the filtrate was measured by an inductively coupled plasma emission spectrometer (ICP-OES, Optima 2000 DV, PerkinElmer). Moreover, similar testing procedures were employed in the test on coexisting ions effect. Using sodium salts such as NaCl, NaNO_3_, Na_2_CO_3_, and Na_2_SO_4_, common anions (Cl^−^, NO_3_^−^, CO_3_^2−^, and SO_4_^2−^) with an exceptionally high concentration of 1 g/L were introduced into the 50 mL solutions (50 ppm arsenate) with the adsorbent dosage of 0.5 g/L at pH 2, in order to investigate the respective influence of these coexisting anions towards the arsenic adsorption process. Furthermore, in the adsorption isotherm study, 0.025 g adsorbent was added to a series of 50 mL arsenate solutions with different initial concentrations from 10 to 200 ppm. Two sets of experiment at pH 2 and 7 were conducted, and the respective solution pH was maintained throughout. Other procedures were the same with those in the pH effect experiment.

## Additional Information

**How to cite this article**: Wang, C. *et al.* Superior removal of arsenic from water with zirconium metal-organic framework UiO-66. *Sci. Rep.* **5**, 16613; doi: 10.1038/srep16613 (2015).

## Figures and Tables

**Figure 1:**
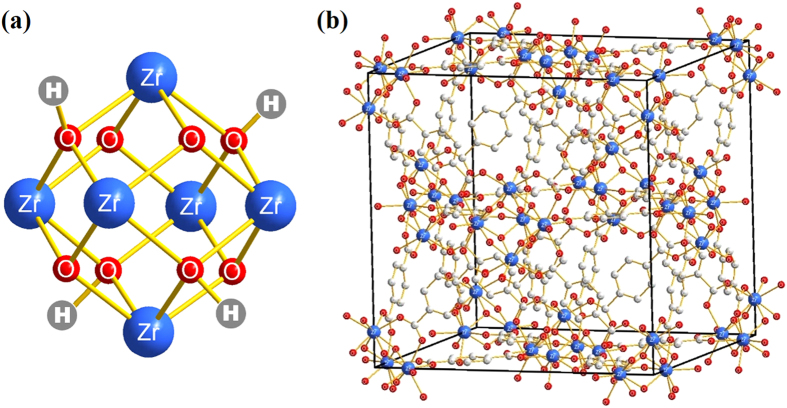
(**a**) Six-centre octahedral zirconium oxide cluster. (**b**) **fcu** unit cell of UiO-66; blue atom – Zr, red atom – O, white atom – C, H atoms are omitted for clarity.

**Table 1:** Langmuir and Freundlich isotherm parameters for arsenate adsorption onto UiO-66 adsorbents, [UiO-66] = 0.5 g/L and T = 25 ± 1 °C.

pH	Langmuir isotherm			Freundlich isotherm		
	q_max_ (mg/g)	b (L/mg)	r^2^	K	n	r^2^
2.0	303.34	6.13	0.92	217.47	9.16	0.83
7.0	147.71	0.42	0.99	62.31	4.74	0.89

**Table 2:** Comparison of arsenate adsorption among prevalent adsorbents.

Adsorbent	Max. adsorption capacity (mg/g)	Working pH range^a^	Ref.
Aluminium-loaded Shirasu-zeolite	5.63 at pH 7	3–10	[10]
Fe-BTC	12.3 at pH 4	2–10	[32]
Commercial TiO_2_	14.2 at optimal pH	Unknown	[8]
Activated alumina grains	15.9 at pH 5	2–7	[9]
MIL-53(Fe)	21.3 at pH 5	3–6	[31]
Activated carbon	30.5 at pH 7	6–8	[6]
Amended Silicate^TM^ adsorbents (ADA Technologies)	40 at pH 7	6–9	[7]
ZIF-8	60 at pH 7	6–8	[30]
Fe–Mn binary oxide	69.8 at pH 5	4–8	[48]
Nanostructured iron(III)-copper(II) binary oxide	82.7 at pH 7	3–7	[49]
Amorphous zirconium oxide nanoparticles	95 at pH 2	2–7	[33]
Zirconium immobilized nano-scale carbon	110 at pH 2	2–6	[34]
γ-Fe_2_O_3_ nanoparticles encapsulated in macroporous silica	248 at pH 6	2–6	[15]
Zirconium based nanoparticle	256.4 at pH 3	2–6	[50]
Yttrium−manganese binary composite	279.9 at pH 7	4–7	[16]
UiO-66	303.3 at pH 2	1–10	This study

^a^Working pH range is defined as the pH conditions at which 60% of maximum adsorption capacity is retained.
